# Ovarian hormones-autophagy-immunity axis in menstruation and endometriosis

**DOI:** 10.7150/thno.55241

**Published:** 2021-01-19

**Authors:** Hui-Hui Shen, Tao Zhang, Hui-Li Yang, Zhen-Zhen Lai, Wen-Jie Zhou, Jie Mei, Jia-Wei Shi, Rui Zhu, Feng-Yuan Xu, Da-Jin Li, Jiang-Feng Ye, Ming-Qing Li

**Affiliations:** 1 Laboratory for Reproductive Immunology, NHC Key Laboratory of Reproduction Regulation (Shanghai Institute of Planned Parenthood Research), Hospital of Obstetrics and Gynecology, Shanghai Medical School, Fudan University, Shanghai 200080, People's Republic of China.; 2 Assisted Reproductive Technology Unit, Department of Obstetrics and Gynecology, Faculty of Medicine, Chinese University of Hong Kong, Hong Kong, People's Republic of China.; 3 Center of Reproductive Medicine of Ruijin Hospital, Shanghai Jiao Tong University School of Medicine, Shanghai 200025, People's Republic of China.; 4 Reproductive Medicine Center, Department of Obstetrics and Gynecology, Nanjing Drum Tower Hospital, The Affiliated Hospital of Nanjing University Medicine School, Nanjing, 210000, People's Republic of China.; 5 Center for Human Reproduction and Genetics, Suzhou Municipal Hospital, Suzhou 215008, People's Republic of China.; 6 Wallace H. Coulter Department of Biomedical Engineering, Georgia Institute of Technology and Emory University, Atlanta 30332, Georgia, USA.; 7 Division of Obstetrics and Gynecology, KK Women's and Children's Hospital, 229899, Singapore.; 8 Shanghai Key Laboratory of Female Reproductive Endocrine Related Diseases, Shanghai, 200080, People's Republic of China.

**Keywords:** autophagy, endometrium, estrogen, macrophage, menstruation, neutrophil, NK cell, progesterone

## Abstract

Menstruation occurs in few species and involves a cyclic process of proliferation, breakdown and regeneration under the control of ovarian hormones. Knowledge of normal endometrial physiology, as it pertains to the regulation of menstruation, is essential to understand disorders of menstruation. Accumulating evidence indicates that autophagy in the endometrium, under the regulation of ovarian hormones, can result in the infiltration of immune cells, which plays an indispensable role in the endometrium shedding, tissue repair and prevention of infections during menstruation. In addition, abnormal autophagy levels, together with resulting dysregulated immune system function, are associated with the pathogenesis and progression of endometriosis. Considering its potential value of autophagy as a target for the treatment of menstrual-related and endometrium-related disorders, we review the activity and function of autophagy during menstrual cycles. The role of the estrogen/progesterone-autophagy-immunity axis in endometriosis are also discussed.

## Introduction

Menstruation refers to cyclical shedding and regeneration of the endometrium occurring in very few species. It is regarded as an inflammatory event regulated by ovarian hormones 1, 2. Menstruation occurs up to 400 times during a woman's reproductive lifespan. Endometriosis is prevalent in 10% of women of reproductive age worldwide, characterized by growth of endometrial tissue outside of the uterus, mainly on the ovaries and the pelvic peritoneum 3, 4. Although the mechanisms of endometriosis remain unclear, retrograde menstruation is known as a key factor in its pathogenesis 5. Likewise, how ovarian hormones orchestrate cyclic changes of endometrium during menstruation has not been fully clarified. Recently, a growing number of studies have reported the crucial role of the interaction between estrogen signaling and autophagy in menstruation as well as endometriosis.

Macroautophagy/autophagy is an intracellular self-digestion pathway for the maintenance of homeostasis. This can be found in all eukaryotic cells at basal level and can be further activated by a variety of stimuli, including deprivation of nutrients, infection, hormones, oxidative stress and endoplasmic reticulum stress 6. It is characterized by the formation of autophagosomes with double-membrane structure, which fuse with lysosomes to degrade cytosolic proteins, damaged or excess organelles, protein aggregates, and invasive microbes 7.

It is well accepted that autophagy plays a critical role in cancers, embryo implantation and reproduction 8-11. Interestingly, increasing evidence indicates autophagy is also involved in menstruation and endometriosis. In this review, we discuss the role of autophagy-immune regulation axis controlled by ovarian hormones in menstruation, its role in the pathogenesis of endometriosis and its potential values in the prevention and treatment of menstrual-related and endometrium-related diseases.

## Classical viewpoint

According to the conventional viewpoint, by exposure to sequential ovarian estrogen and progesterone will cause the endometrium to undergo well-defined cycles of proliferation, differentiation, and shedding (menstruation) on a monthly basis 12, 13. The molecular mechanisms underlying these events involves complex crosstalk between the endocrine and immune system 13, 14. In the absence of pregnancy, progesterone withdrawal drives a local inflammatory response in the uterus involving infiltration of leukocytes, release of cytokines, activation of matrix metalloproteinases (MMPs) and disintegration of extracellular matrix (ECM) 12, 15. The presence of progesterone inhibits neutrophils entry into the endometrium 14, NF-κB activation, and the expression of several stromal cell-derived, lytic enzymes, including urokinase-type plasminogen activator (uPA), MMP-1, and MMP-3 16. Thus, withdrawal of progesterone can be followed by a dramatic rise in the endometrial leukocyte population. Besides on the generation of more cytokines (e.g. interleukin (IL)-1, IL-6) and proteases (e.g. MMPs), these cells are also involved in ECM breakdown and debris removal.

Among the inflammatory mediators stimulated, IL-8 [a neutrophil chemotactic factor, also called C-X-C motif chemokine ligand 8 (CXCL8)], monocyte chemoattractant protein-1 [MCP-1, also called C-C motif chemokine ligand 2 (CCL2)] and cyclooxygenase-2 [COX-2, the inducible enzyme responsible for synthesis of prostaglandins (PGs)], are well-studied 17. COX-2 upregulation mediated by nuclear factor kappa-B (NF-κB) leads to increased levels of PGs. PGF2α causes myometrial contractions and vasoconstriction of the endometrial spiral arterioles, resulting in a hypoxic environment in the endometrium 18.

Hypoxia during menstruation can directly upregulate vascular endothelial growth factor (VEGF) expression in endometrial stromal cells (ESCs) by modulating the NF-κB pathway 19. VEGF is an important regulator of angiogenesis that stimulates the proliferation, migration, and proteolytic activity of endothelial cells. It can also induce MMP-1, -3 and -9 expression in vascular smooth muscle cells, which is required for tissue repair. Following endometrial breakdown, MMP activity can be inhibited by tissue inhibitors of metalloproteinases (TIMPs) or by the protease inhibitor α2-macroglobulin, which can ensure the limitation of tissue damage. During the proliferative phase, the functional layer of the endometrium experiences a rapid growth under the influence of estrogen. As a result, concomitant angiogenesis occurs to repair ruptured blood vessels and maintains perfusion of the new tissue.

## Autophagy-related molecules during the menstruation cycle

### Autophagy-related genes and molecules in the endometrium

Autophagy involves five steps, namely, nucleation, elongation, maturation, fusion and degradation by lysosomes. Double-membraned structures first engulf portions of the cytoplasm to form autophagosomes. Autophagosome is initiated by the unc-51-like kinase 1 (ULK1) complex (including ULK1, ULK2, Atg13, FIP200 and Atg101). This complex can be suppressed via mammalian target of rapamycin complex (mTOR). During autophagosomal vesicle elongation and maturation, autophagy-related (Atg) protein 7 and Atg10 promote Atg12 and Atg5 conjugation. Such that, Atg 12 and Atg5 could interact with Atg16L1 to form a dimeric complex (Atg12-Atg5-Atg16L1), which is required for formation of the covalent bond between LC3 and phosphatidylethanolamine (PE). Then LC3 precursor (pro-LC3) converts into LC3-Ι in the action of Atg4, which is activated by Atg7 and transferred to Atg3. The Atg12-5-16L1 complex facilitates the transfer of LC3-Ι from Atg3 to PE to generate microtubule-associated protein 1 light chain 3 (LC3‑II) 20, which is a standard marker of autophagic flux and localizes to both the inner and outer autophagosomal membranes.

LC3-Ι, a marker of autophagy, is increased during autophagy induction, both in endometrial glandular epithelial cells (EPCs) and ESCs throughout the menstrual cycle. LC3-I expression is very low in the proliferative phase, but it surges to peak level at the late secretory phase 21. LC3-II expression in the late proliferative phase is higher than in the early proliferative phase 22. Additionally, autophagosome numbers are higher in secretory ESCs, compared with the proliferative phase 23.

### Role of ovarian hormones on endometrium autophagy

#### Effects of ovarian hormones on endometrial autophagy

**Figure 1:**
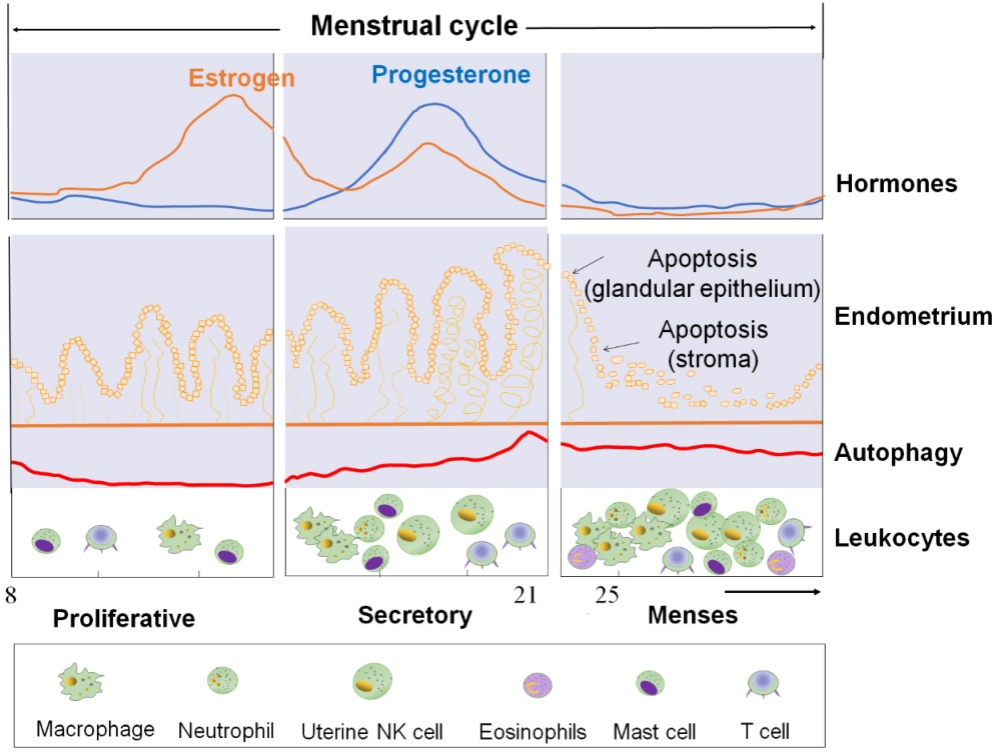
** The cyclical changes of ovarian steroid hormones, endometrium, and autophagy level in ESC and autophagy levels of ESC and the infiltration of immune cells during the menstrual cycle.** First panel: Estrogen and progesterone levels throughout the menstrual cycle. Second panel: The morphological changes in the endometrium during the menstrual cycle. Third panel: Autophagy during the menstrual cycle. Bottom panel: Trafficking of Leukocytes into the endometrium during the menstrual cycle. The size of the cells represents the abundance. ESC: endometrial stromal cell.

During the normal human secretory phase ESCs, estrogen is a potent suppressor of autophagy while progesterone can significantly stimulate the activities of autophagy 23, 24. During menstruation cycle, autophagy levels remain low in the proliferative phase but significantly increase at the late secretory phase (**Figure 1**) 23, 24. Choi *et al*. showed that the expression levels of LC3-II and cleaved caspase-3 increased significantly in normal ESCs cultured with estrogen and progesterone when compared with those cultured with estrogen alone 22.* In vitro* experiment also reported the autophagy level in endometrial cells treated with estrogen alone (*in vitro* imitation of the proliferative phase) was low and was upregulated after the supplementation with progesterone (*in vitro* imitation of early-to-mid secretory phase) or following the removal of estrogen and progesterone (*in vitro* imitation of late secretory and menstrual phases) 25. Specifically, progesterone significantly increased LC3-II and ATG5 expression in estrogen-treated normal ESCs, while AKT phosphorylation (which negatively regulate autophagy induction by activating mTOR) was significantly decreased 25. This change could be suppressed by the addition of mifepristone (progesterone receptor modulator) 25. Notably, the stimulatory effect of progesterone on ESC autophagy was also dependent on the CXCL12/CXCR4 axis 22.

In addition to ESCs, a large number of EPCs also reside in the endometrium, especially during the secretory phase of the menstrual cycle. The regulation of autophagy in normal EPCs by ovarian steroids has not been reported so far, which may be due to the fact that the primary normal EPCs are very difficult to obtain and culture. Nevertheless, some insights can still be obtained from studies on autophagy in uterine corpus endometrial carcinoma (UCEC) cells. Consistent with estrogen's inhibitory effect on the autophagy of ESCs, our previous studies also show that estrogen significantly restricts autophagy of UCEC cells *in vitro* and *in vivo* 26, 27. Many drugs targeting autophagy-suppression are already in clinical use for endometrial hyperplasia 28. Additionally, protopanaxadiol (PPD) and metformin alone or in combination exert their therapeutic roles by inducing autophagy of UCEC cell with higher levels of Beclin1 and LC3 II/LC3 I, and low level of p62 expression 27. These reports demonstrate that estrogen has a strong inhibitor effect on the autophagy of ESCs and EPCs. Admittedly, there are some inconsistent results in other types of cells due to different local microenvironments 29-31.

Therefore, it may be hypothesized that, with estrogen and progesterone withdrawal, there should be higher levels of autophagy in ESC and EPC due to the absence of powerful estrogen-mediated autophagy inhibition (**Figure 1**).

#### How do ovarian hormones affect endometrial autophagy?

Ovarian hormones, that are the upstream of signaling mediators to regulate various aspects of uterine physiology, also serve as regulators of autophagy. Estrogen may mediate autophagy through its receptors (ER), namely ERα, ERβ and G-protein coupled estrogen receptor (GPER) 31, 32. In both the human and nonhuman primate endometrium, ERα and ERβ are expressed in the nuclei and cytoplasm of glandular epithelial and stromal cells, which decline in the mid secretory phase in the functional layer 33. Endometrial ERα levels are upregulated during the proliferative phase by estrogen, which is at its peak during the mid- to late proliferative phase of the menstrual cycle 34, and subsequently is downregulated in the secretory phase by progesterone 35, 36. Estrogen suppresses autophagy through ERα in neurons, which contributes to less severity of iron-induced brain injury women compared with that in men 37. Our previous research showed that estrogen could suppress autophagy through CXCL12/CXCR4 interaction and NF-κB signal activation in ESCs 23. However, PPD downregulates the expression of ERα, upregulates the expression of progesterone receptors (PGRs), which restricts estrogen-mediated anti-autophagy effects in ESCs 38. Moreover, fulvestrant is a selective ER antagonist that can abrogate the inhibitory effect of estrogen on autophagy in UCEC cells 26.

According to a number of recent studies, progesterone and progestins can be particularly important as inhibitors of mTOR in various cell types 25, 39, which can result in the activation of autophagy. These effects are mediated by PGR, consisting of PGRA and PGRB. Progesterone can activate PGRB and further induce autophagy via mediating nuclear translocation and activation of transcription factor EB (TFEB) 40. These can then act as a master transcriptional regulator that would control the expression of autophagy and lysosomal genes. Therefore, TFEB upregulates the expression of autophagy and lysosomal genes to activate autophagy. Moreover, inhibition of progesterone receptor membrane component 1 (PGRMC1, also known as sigma-2 receptor) can increase the activation of adenosine 5'-monophosphate (AMP)-activated protein kinase (AMPK) and promotes the levels of tuberous sclerosis complex (TSC), which causing the inactivation of mTOR and the elevated autophagic flux 41. Combined with progesterone, PGR can activate MAPK/ERK and the transcription factor cyclic AMP response-element binding protein (CREB), thereby modulating the expression of genes required for cell proliferation, inflammatory responses, differentiation, and apoptosis 42.

**Figure 2:**
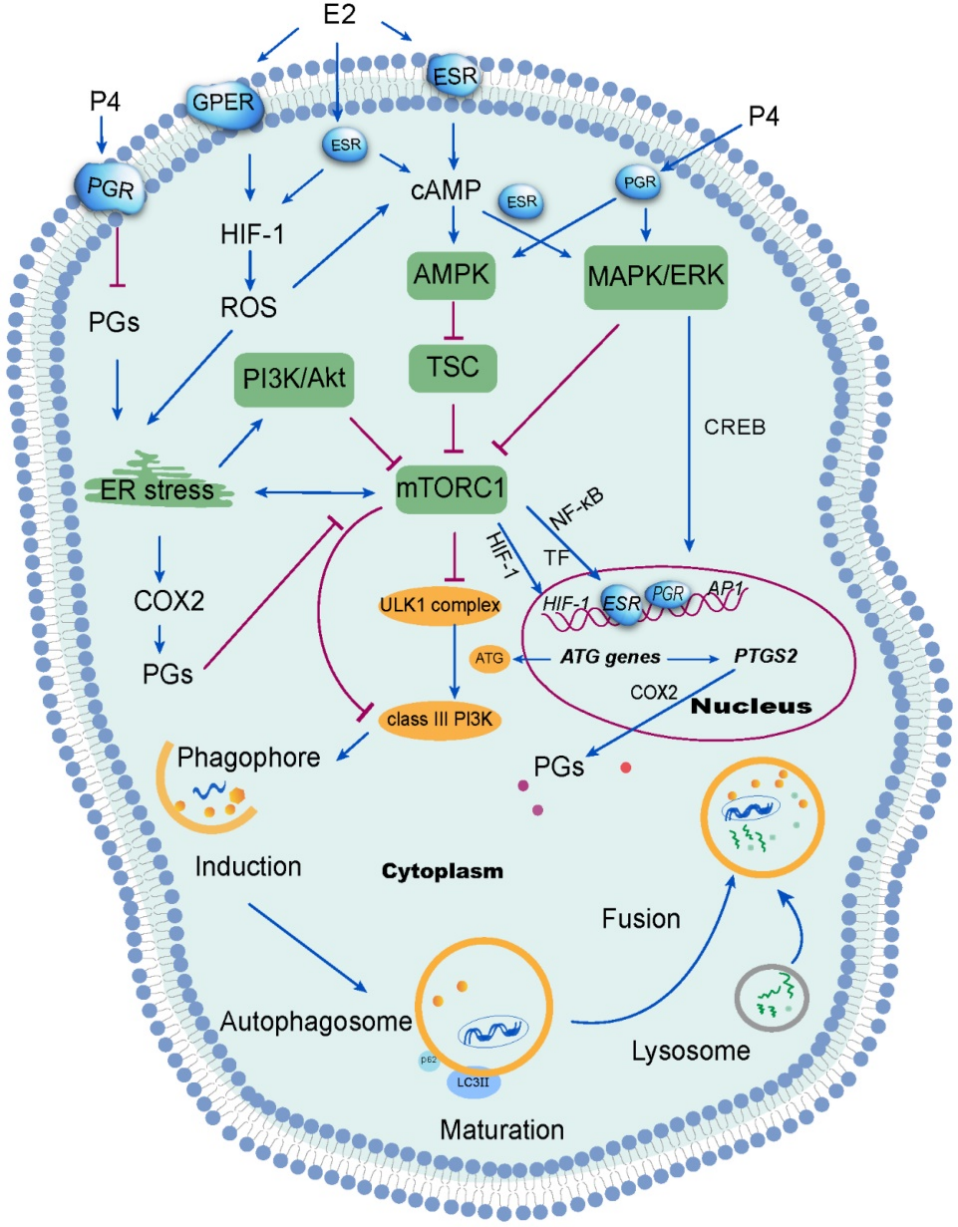
** Signal transduction mechanisms activated by ovarian steroid hormones on endometrial autophagy**. Estrogen (E2) inhibits reactive oxygen species (ROS) production, which further suppresses autophagy in the endometrium. ROS has been shown to suppress the mTOR signaling pathway and further induce autophagy through several mechanisms, including activation of endoplasmic reticulum (ER) stress. Additionally, ER stress induces autophagy via the PI3K/AKT pathway and the COX-2/prostaglandins (PGs) axis. Of note, progesterone (P4) suppresses ER stress by restricting the PGF2α/ROS axis. However, the inhibition of mTOR signaling aggregates ER stress. Under stimulation from estrogen or progesterone, autophagy is induced through the activation of the MAPK/ERK pathway, which regulates autophagy-related genes through the transcription factor* CREB*. Therefore, the withdrawal of estrogen and progesterone leads to the inactivation of mTOR signaling and high levels of endometrial autophagy during menstrual phase through the HIF-1/ROS/AMPK signaling pathway, ER stress and MAPK/ERK signaling pathway.

Hypoxia-inducible factor-1 (HIF-1), a transcription factor which regulates cellular response to hypoxia, is expressed exclusively during the secretory and menstrual phases, suggesting hypoxia would occur in the endometrium late in the menstrual cycle 43. It has been reported that estrogen negatively correlatives with HIF-1 levels in other cells (e.g, macrophages and breast cancer cells) 44, 45. Following treatment with follicle-stimulating hormone (FSH), autophagy signaling was activated in mouse granulosa cells via HIF-1α 46, contributing to follicle development and atresia. Estrogen withdrawal is linked to endometrial hypoxia during menstruation when spiral arterioles contract under the action of PGF_2_. Notably, overloaded reactive oxygen species (ROS) are generated due to the lack of oxygen, nutrient starvation, mitochondrial toxins and oxidative stress, resulting in organelle damage and the production of toxic substances 47, 48. ROS elicits autophagy by reducing the ATP (adenosine triphosphate)/AMP (adenosine monophosphate) ratio. As a result, AMPK activates the TSC, leading to mTOR inactivation and initiation of autophagy 49. Studies have shown that estrogen can inhibit ROS production, thus protecting cells from oxidative stress damage 50, 51. Interesting, ERβ and GPER have also been reported to be involved in this process 50, 52. Sugino *et al*. have reported that estrogen and progesterone withdrawal stimulated PGF2α production through ROS-induced NF-κB activation in human ESCs 53. From these reports, it can be concluded that estrogen withdrawal might induce autophagy of endometrium during menstruation through the activation of the HIF-1/ROS/AMPK signaling pathway and further inactivation of mTOR signaling (**Figure 2**).

Several studies have identified the AMPK/TSC/mTOR signaling pathway as the classical signaling pathway regulating autophagy 54. In mammals, various stimuli, including hypoxia, activate the AMPK or mitogen-activated protein kinases (MAPK)/ERK (extracellular signal-regulated kinases) signaling pathways can result in the suppression of mTOR and the activation ULK1 complex 55. Alternatively, the inhibition of class I phosphatidylinositol 3-kinase (PI3K) and its downstream target AKT suppresses mTOR signaling. In turn, the inhibition of mTOR reduces the phosphorylation of ATG13 56. Thereafter, ATG13 forms a ULK1 complex with ULK1, focal adhesion kinase family interacting protein of 200 kDa (FIP200) and ATG101, to induce autophagy by phosphorylating Beclin 1 and activate class-III PI3K complex (hVps34-hVps15-Beclin1) 57. Under nutrient-rich conditions, the mTOR complex 1 (mTORC1) mediates phosphorylation-dependent inactivation of the kinase activities of ATG13 and ULK1, leading to the inhibition of autophagy. In the event of starvation or treatment with rapamycin, mTORC1 will dissociate from this complex to decrease mTORC1-mediated phosphorylation of ATG13 and ULK1. During the initiation of autophagy, ATG7 (E1 ubiquitin-activating enzyme-like) activates the ubiquitin-like protein ATG12. Activated ATG12 subsequently is transferred to ATG10 (E2 ubiquitin-conjugating enzyme-like) and is ultimately linked covalently to ATG5 to form a complex (E3-like) with ATG16L1 58. This large complex is vital for the elongation of phagophores, but dissociates after autophagosome formation 59. In addition, the LC3 precursor undergoes C-terminal cleavage by ATG4B to produce LC3-I. LC3-I is then conjugated to PE via interaction with Atg7 (E1-like), Atg3 (E2-like), ATG16L1 complex in sequence, generating LC3-II. Previous studies indicated that ovarian hormones (estrogen and progestogen) promoted breast tumorigenesis by enhancing insulin like growth factor receptor (IGFR) and Akt/mTOR signaling to inhibit apoptotic stimuli 60, suggesting that the suppression of autophagy induced by estrogen in breast cancer cells could be abolished by silencing ERα or knocking out ATG7 37. Akt is considered as a survival factor of endometrial cells in rodents 61. Rapid activation of Akt by estrogen and progestogen has also been shown in human ESCs, resulting in proliferation and migration of ESCs 24, 61. Moreover, phospho-Akt levels decreased in human ESCs when decidualization was induced triggered by high levels of progesterone 62. These reports suggest that the Akt/mTOR signaling pathways could be involved in the induction of autophagy in the endometrium, due to estrogen and progesterone withdrawal during the menstrual phases (**Figure 2**). However, the specific mechanism has yet to be clarified.

In response to progesterone, endometrial stromal fibroblasts acquire epithelioid characteristics, such as a highly developed endoplasmic reticulum required for greater secretory function, substantial lysosomes, abundant glycogen and lipids droplets in their cytoplasm 63. In animal models, the expression of endoplasmic reticulum stress-related proteins and the autophagy marker LC3-II are increased in luteal cells treated with PGF2α 64. Physiologically, elevated PGF2α in menstruation induces endoplasmic reticulum stress. Consequently, autophagy is induced by endoplasmic reticulum stress, with the activation of inositol-requiring protein-1α (IRE1α), protein kinase RNA (PKR)-like ER kinase (PERK), and activating transcription factor 6 (ATF6) 65, 66. The activation of PERK inhibits general protein translation by inducing eIF2α phosphorylation, enabling translation of activating transcription factor 4 (ATF4) 67. ATF4 can activate autophagy via the induction of several ATG genes 68. For example, endoplasmic reticulum stress eIF2α-ATF4 pathway-mediated COX-2 overexpression contributes to kidney autophagy and injury 69. It has been reported that progesterone has an inhibitory effect on PG secretion of pig endometrial glandular and stromal cells *in vitro* 70. In addition, progesterone can alleviate ROS stress in UCEC cells 71. Therefore, it can be speculated that progesterone prevents endometrial cells from endoplasmic reticulum stress by controlling the PGF2α/ROS signaling pathway. The withdrawal of progesterone may participate in the induction of high autophagy in endometrium and menstruation through the regulation of endoplasmic reticulum stress and these downstream metabolic signaling pathways. Further studies are needed to clarify the detailed mechanisms.

## The estrogen / progesterone-autophagy-immunity axis in menstruation

### Infiltration of immune cells in endometrium and immune defense

Under the cyclic regulation of estrogen and progesterone, leukocytes populations within the endometrium vary considerably across the menstrual cycle, and the mass infiltration of immune cells is closely associated with embryo implantation and menstruation 72. During the mid-secretory phase of the human menstrual cycle, a large increase of specific subpopulations of immune cells [mainly uterine NK (uNK) cells and macrophages] infiltrate the endometrium to modulate uterine receptivity and embryo implantation 72. In particular, many chemokines (e.g, CXCL12, CCL2, CCL4 and CX3CL1) are involved in these processes 23, 72. With the withdrawal of estrogen and progesterone, however, the increase of COX-2 and PGF2α promotes uterine contractility and constricts the spiral arterioles in the late secretory phase endometrium. Subsequently, inflammatory responses and the formation of a hypoxic microenvironment further induce an influx of more immune cells (predominantly macrophages, uNK cells, neutrophils, eosinophils and mast cells) by the upregulation of chemokines and their receptors, including CXCL8 and CCL2, eotaxin, MMPs, and CX3CR1 (**Figure 1**) 72. These immune cells play an important role on the immunity during the menstrual period.

It has been reported that adipose mTORC1 suppresses PGs signaling and beige adipogenesis via the CREB-regulated transcription coactivator 2 (CRTC2)-COX-2 pathway 73. Such that, ATG7 regulates ultraviolet-induced cytokine expression and secretion, which can promote COX-2 expression in keratinocytes in a CREB1/CREB-dependent and IL1β-dependent manners 74. In addition, autophagy promotes CCL2 transcription in epidermal keratinocytes through the AMPK-BRAF-MAPK1/3/ERK-activator protein 1 (AP1) pathway 75, and upregulates CXCL8 to trigger mesenchymal stem cell-mediated CD4^+^T cell migration and differentiation 76. The presence of microbes in the uterine cavity has been widely demonstrated, which initiates immunologic reactions 77. High levels of autophagy may participate in the regulation of endometrial infiltration of immune cells to play a protective role during the menstrual cycle, and the activation of COX-2/PGE_2_ and up-regulation of chemokines may be involved in these processes (**Figure 2**).

Macrophages are present throughout the menstrual cycle but display a substantial increase in number during the perimenstrual phase in response to progesterone withdrawal 78. After macrophage infiltration, other immune cells, which are critical in cell-mediated immunity and in the resolution of inflammation will further be recruited 79. Evidence shows that induction of autophagy is critical for the survival and differentiation of monocytes 80. Under the stimulation of colony-stimulating factor 1 (CSF1) or granulocyte-macrophage colony-stimulating factor (GM-CSF), macrophages will be differentiated from monocytes. These processes are initiated dependently by the release of Beclin1 from Bcl2 from activated c-Jun N-terminal kinase (JNK) and blockage of ATG5 cleavage 80, 81. Additionally, ROS production can be induced by CSF2, which is a potent stimulator of autophagy and persists in the uterus during the secretory phase 82. In addition, estrogen can reduce the expression of HIF-1α in macrophages during the proliferative phase 45. Therefore, autophagy level of macrophage should be elevated during menstrual phase. Adequate autophagy allows macrophages to maintain immune homeostasis and function, for pattern recognition, cytokine release, inflammasome activation, and LC3-associated phagocytosis 83, 84. Hence, this making COX-2 an enhancer of the anti-microbial function of macrophages through the activation of autophagy 17, 85.

Typically, NK cell numbers increase dramatically from post-ovulation (around LH+3) to few days prior to menstruation 72. As found, uNK cells (CD56^bright^CD16^-^), which is usually found near the endometrial glands and spiral arteries, increase in number from the proliferative phase and peak in the late-secretory phase 86. These processes are mainly due to the recruitment of high levels of CXCL12 in the endometrium, which is modulated by estrogen and progesterone 23. The cytotoxicity of uNK cells with high levels of the activation markers (CD69 and HLA-DR) is, comparable to peripheral NK cells in the late-proliferative phase. These characteristics of uNK cells are likely to prevent microbial infections 77. Our recent studies demonstrated that the increase of autophagy of ESCs should promote the residence of NK cells in endometrium/decidua through upregulating MMP-9 and adhesion molecules expression 87. Therefore, the increased numbers of resident uNK cell in the endometrium, triggered by high levels of autophagy during menstruation, supports immune defense.

During the early proliferative phase of the menstrual cycle, neutrophils and eosinophils are scarce in the human endometrium. However, their numbers significantly increase when reaching into premenstrual period 77. Mast cells, located in close proximity to vessels, the neutrophil chemoattractants CXCL1 and CXCL2, which then works with macrophages 88 to recruit neutrophils 89. LC3-II is mainly localized in the secretory granules of mast cells. Besides, autophagy is involved in the degranulation of mast cells, consolidating the antimicrobial capacity 90. Such that, inhibition of autophagy or deficiency of either ATG5 or ATG7 was shown to reduce the degranulation of neutrophils in mouse models 91. Importantly, the presence of autophagy in neutrophils is beneficial for bacteria clearance 91 and act as a modulator for multiple functions, including phagocytosis, degranulation, ROS production, formation of NET, and IL-1β production 92-94. During menstruation, therefore, the increase in uterine contractility and local hypoxia should trigger autophagy in neutrophils and mast cells in the endometrium to act as a beneficiary for fighting various pathogens within uterus.

### Removal debris and dead cells during menstruation

The spontaneous periodic apoptosis during the menstrual cycle is essential for maintaining the normal structure and function of the endometrium, while cell autophagy plays a critical role in the apoptosis of human endometrial cells at different phases of the menstrual cycle 95, 96. High levels of autophagy in the endometrium during the mid- and late secretory stage can trigger autophagic cell death and initiate apoptosis to prepare for endometrial exfoliation during menses, this may then contribute cyclic remodeling of the endometrium 95, 97. Coincident with changes in autophagic activity, cleaved caspase 3 expression levels in glandular epithelial cells increase significantly during the secretory phase, reaching a maximum during the late-secretory phase 95. In addition, autophagy induces G0/G1 arrest and apoptosis in menstrual blood-derived endometrial stem cells 97. Autophagy of ESC promotes the upregulation of MMP9 in a melanocyte inducing transcription factor (MITF)- herpesvirus entry mediator (HVEM)-dependent manner 87, resulting in rapid ECM degradation and endometrial exfoliation occur during menstruation. Remarkably, the intracellular composition of the ESC can be renewed through autophagy. This is brought upon by the rapid clearance of mRNA and new proteins synthesized by the gene expression program, as well as the recycling of damaged organelles 98.

**Figure 3:**
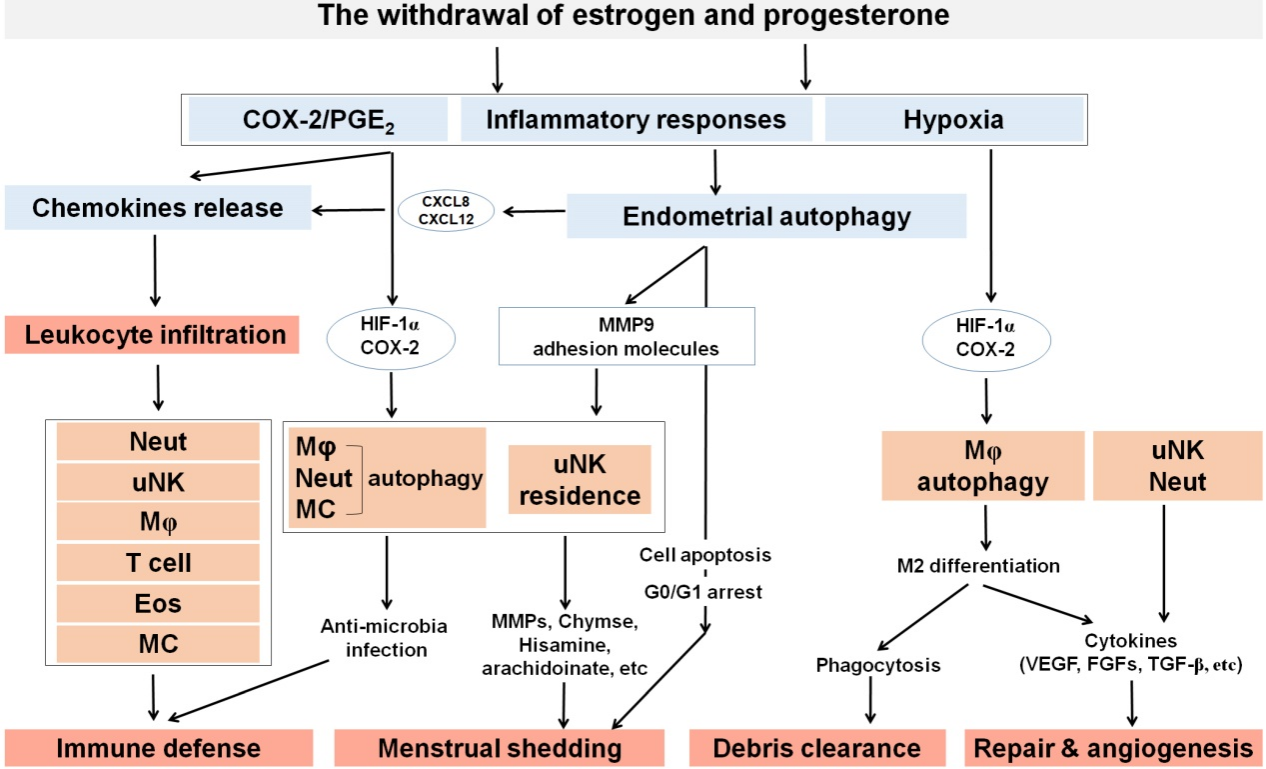
** The roles of ovarian steroid hormones-autophagy-immunity axis in menstruation.** With the withdrawal of estrogen and progesterone, local inflammatory responses are initiated in the endometrium together with the activation of endometrial autophagy. Subsequently, MMPs are released and synergize with other inflammatory factors to participate in menstrual shedding. The release of a large number of chemokines (e.g., CXCL8, CXCL12, CCL2, CCL4 and CX3CL1) results in leukocyte infiltrations (neutrophils, uterine NK cell, macrophage, T cell, eosinophils, and mast cells) that can contribute to immune defense during menstruation. Residence of uterine NK cell (uNK) activated by endometrial autophagy and mast cells accelerate menstrual shedding by MMP9, Chymase, and etc. Additionally, elevated levels of COX-2 and PGs not only generate a hypoxic environment, but also induce autophagy in macrophages, neutrophils and mast cells that protect the endometrium from infections. COX-2 is also produced as a result of hypoxia during late menstruation, facilitating M2 macrophages polarization which participates in debris clearance, endometrial repair and remodeling. Meanwhile, uNK and neutrophils also contribute to endometrium repair and remodeling by secreting various cytokines (e.g, VEGF, FGF, and TGF-β). Neut: neutrophils; Mφ: macrophage; EOS: eosinophils; MC: mast cell.

Under the regulation of endometrial autophagy, a large number of infiltrating leukocytes accelerate endometrial exfoliation and become the main force for removing cellular debris, apoptotic and necrotic cells (**Figure 3**). Among these, macrophages and uNK cells secrete MMP-7 and MMP-9, resulting in the apoptosis of smooth muscle cells and endothelial cells 99. The proinflammatory cytokines secreted by these immune cells, such as TNF-α, play a role in menstrual shedding, by upregulating the expression of Fas and facilitating Fas-mediated apoptosis 100. Mast cells in endometrium express tryptase and chymase, likely contributing to menstruation. Chymase, in particular, can play an important role in establishing a cascade of MMPs activation 77. In addition, mast cells produce histamine, arachidonate, heparin products and a large number of pleiotropic cytokines. They have important effects on endothelial cell function and local induction of edema. More importantly, CD68^+^ macrophages are abundant in the human endometrium during tissue breakdown and repair. As scavenger cells, macrophages can eliminate apoptotic cells in the uterus and endometrial cells in the peritoneal cavity following the retrograde flow of menstrual blood 101. Certainly, this process is dependent on the bioavailability on autophagy of macrophages.

### Endometrial tissue remodeling during late menstruation

A high level of autophagy during late menstruation facilitates M2 macrophages polarization to participate in efficient repair and remodeling (**Figure 3**) 102, 103. It is well established that classically activated “M1” macrophages produce inflammatory cytokines while the activated “M2” macrophages (CD68^+^/CD163^+^) promote tissue remodeling and anti-inflammatory reactions due to reduced proinflammatory cytokine secretion and increased endocytic clearance capacity 104, 105. Endometrial macrophages constantly change their phenotype to adapt to their microenvironment 78. Macrophages at wound site express transforming growth factor (TGF) α, fibroblast growth factors (FGFs), platelet-derived growth factor (PDGF), VEGF and TGFβ, leading to rapid removal of cell debris and replacement by granulation tissue containing inflammatory cells and blood vessels 106. As reported, the depletion of macrophages at other sites of injury results delayed and defective repair 106. High levels of VEGF induced by hypoxia during menstruation promote M2 macrophage recruitment, migration and polarization by the VEGFR1/VEGF axis 107. Besides, MMP-27 is mainly expressed by M2 macrophages in normal endometrium throughout the menstrual cycle and in endometriotic lesions. This plays a critical role in the regeneration of the endometrial epithelial lining and angiogenesis 108-110. Wang *et al*. demonstrated that the hypoxia-autophagy axis induced VEGFA expression in peritoneal mesothelial cells 111, which suggests that the hypoxia-autophagy axis could be involved in endometrial tissue remodeling by triggering VEGFA-mediated M2 macrophage differentiation and vascular remodeling.

Additionally, the function of neutrophils and uNK cells cannot be ignored. It has been reported that neutrophil depletion with the antibody RB6-8C5 not only affected endometrial breakdown, but also can remarkably delayed endometrial repair 112, highlighting their importance in the destruction of endometrial tissue and concomitant repair. Further, neutrophils promote cyclical endometrial vascular proliferation by expressing VEGF but do not express ERα nor PGR 113. uNK cells may initiate arterial remodeling and maintain vascular stability as they are a rich source of many different cytokines and growth factors including TNF-α, IL-10, GM-CSF, IL-1β, TGF-β1, CSF-1, leukemia inhibitory factor (LIF) and IFN-γ 114. Moreover, Xu *et al*. have reported that the LKB1/p53/TIGAR/autophagy-dependent VEGF expression contributes to pulmonary inflammatory responses 115. Additionally, autophagy has been implicated to cause tumor resistance to anti-angiogenic therapy 116, which is probably dependent on the dual roles of the AMPK/autophagy in angiogenesis 117. Collectively, autophagy affects the function of neutrophils and NK cells, indicating their participation in tissue remodeling.

## Ectopic endometrium autophagy on endometriosis

### Menstruation and endometriosis

Endometriosis is characterized by implantation and growth of endometrial tissue outside the uterine cavity, affecting 10% of women in their reproductive life 3. It has been proposed that endometrial fragments reaching the pelvis via retrograde menstruation, as well as their subsequent adhesion and implantation onto the peritoneum and abdominal organs, resulting in chronic pelvic pain and infertility. However, the pathogenesis and etiology of endometriosis remains unclear. There is increasing evidence that menstruation is linked with this condition. Firstly, this only occurs in women and menstruating primates. Secondly, there are various risk factors for this disease, including early menarche and nulliparity, and sexual activity leading to orgasm during menstruation, which further emphasizes that menstruation may play a key role toward the origin of endometriosis 118, 119. Furthermore, patients with endometriosis have shorter menstrual cycle intervals, heavier menstrual effluent 120 and increased duration of menstrual flow than those without 121. In addition, evidence from animal models showed that intra-peritoneal inoculation with menstrual in baboons resulted in the development of endometriosis 122. Thus, it suggests that retrograde-menstruation is closely linked with the occurrence of endometriosis.

### The link between autophagy in menstruation and endometriosis

Endometriosis is characterized by estrogen dependence and progesterone resistance 123. With the upregulation of 17β-hydroxysteroid dehydrogenase-1 and aromatase genes, the level of estradiol in endometriotic lesions is higher than normal endometrium 124.This increased estradiol would bind and further activate ERs in endometriotic lesions, specifically ERβ 125. A previous report also identified an epigenetic defect involving hypomethylation of a CpG island occupying ERβ promoter, which was found to cause the enhanced expression of ERβ 126. Pathologically high ERβ levels repress ERα and PGR by negatively on regions on their promoter 33. In turn, the reduced PGR activity further promotes estrogen synthesis. Of note, ERβ may upregulate PGE2 in endometriotic cells via the induction of COX2 expression 33. Increased PGE2 induces the production of estradiol 127, 128. As ERα mainly promotes angiogenesis, ERβ has a predominant role in anti-apoptosis, activation of the inflammasome and invasion of ectopic lesions 129, 130. Therefore, excessive estrogen and PGE2 entail inflammation, immune responses, angiogenesis, and promotes the survival of endometriotic lesions 131, 132.

Far beyond that, high estrogen levels and progesterone resistance are also considered to be the regulators on the decreased autophagy in patients with endometriosis 133. When compared with normal ESCs 134, the secretory ectopic and eutopic ESCs in endometriosis patients exhibited decreased expression patterns of autophagy-related genes, mRNA and proteins 135. The difference is even greater when endometriotic tissues are compared with eutopic endometrium 136. Our previous work has identified autophagy-related genes, including *SNCA*, *RGS19*,* IGF1*,* ATG9B*,* ATG12*,* ATG10*,* IFNG*,* PIK3CG*, and *DAPK1*, with lower expression levels in eutopic and ectopic secretory phase ESCs than normal ESCs. Moreover, the genes involved in autophagy initiation and regulation, such as *CXCR4*,* ESR1*, and *mTOR*, are up-regulated, while *ATG16L1*, *IRGM* and *ULK1* are further weakened in ectopic ESCs 23. Choi et al. demonstrated that progesterone had no significant effects on AKT, mTOR activity, autophagy or apoptosis in endometriotic cyst stromal cells. While for endometriotic tissues, they are noticed to have constant autophagy and apoptosis throughout the menstrual cycle 25. These phenomena suggest that the dysregulation of autophagy levels in response to progesterone may play a role in the pathogenesis of endometriosis. For epithelial cells from endometriotic tissues, it has also been shown that both CXCR4 and CXCL12 are higher when compared with normal uterine endometrium 137. Thus, high concentrations of estrogen may contribute to lower autophagy levels of ectopic ESCs and epithelial cells through the activation of the CXCL12-CXCR4 axis 23. In addition, elevated levels of mTOR and the decreased ratio of LC3-II/LC3-I ratio in the eutopic ESCs have been observed 138, 139. It is widely accepted that endometrial cell autophagy exerts an active role in the regulation of apoptosis 140. Reduced autophagy induction via AKT/mTOR signaling and decreased apoptosis were observed during the late secretory phase of the menstrual cycle in endometriotic tissues 25.

By contrast, treatment with PPD markedly reverses the inhibitory effect of estrogen on ectopic ESC autophagy by upregulating PGR and downregulating ER. Moreover, the cytotoxic activity of NK cells in ectopic ESCs was found to be enhanced 38. Taken together, the autophagy levels in patients with endometriosis are strongly reduced due to overexpression of estrogen through complex signaling pathway compared with normal endometrium.

**Figure 4:**
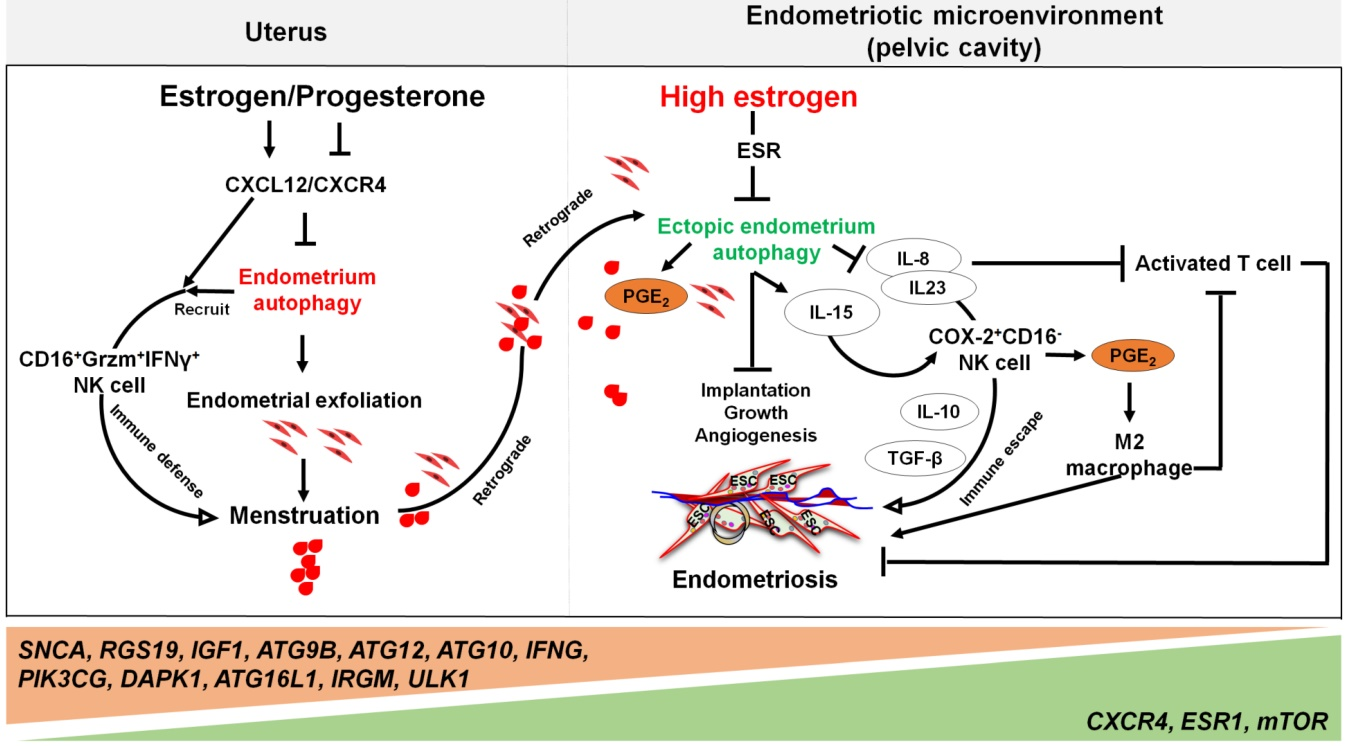
** The involvement of the ovarian steroid hormones-autophagy-immunity axis in the pathogenesis of endometriosis.** In physiological situation, under the periodic regulation of estrogen and progesterone, autophagy levels in the endometrium also change in a CXCL12/CXCR4 dependent manner. During the menstrual period, the withdrawal of estrogen and progesterone leads to high levels of endometrial autophagy, contributing to endometrial exfoliation, immune defense, menstrual debris and endometrial fragment clearance by CD16^+^Granzyme B (Grzm)^+^INF-γ^+^ NK cells. In the presence of high concentrations of estrogen and progesterone resistance, however, autophagy-related genes (e.g., *SNCA*, *RGS19*, *IGF1 ATGs*, *CXCR4*, *ESR1*, and *mTOR*) are altered, leading to decreased endometrial autophagy. The suppression of endometrium autophagy directly accelerates the implantation, growth and angiogenesis of endometriotic lesions, whilst promoting the immune escape of endometriotic lesions through IL-8 and IL-23-mediated COX-2^+^CD16^-^NK cell differentiation. IL-15 is also involved in this process. Additionally, IL-8 inhibits activated T cells, and COX-2-induced PGE2 release stimulates M2 macrophage differentiation, which can facilitate the immune escape, remodeling of ECM and neovascularization of endometriotic lesions. Therefore, the effects of aberrant ovarian steroid hormones-autophagy-immunity axis contribute to the occurrence and development of endometriosis.

Yet, the reduced autophagy and abnormal immune responses in endometriosis are not conducive to the removal of endometrial debris due to impaired immune functions. As previously mentioned, NK cells are involved in the removal of menstrual debris and endometrial fragments that are likely to reach the peritoneal cavity by retrograde menses. Differing from uNK cells in normal endometrium increased remarkedly from the proliferative phase with highest levels in the late secretory phase, uNK cells in ectopic lesions remained significantly low throughout the cycle 86, and allowing refluxed uterine cells to survive in ectopic lesions. Many cells secrete IL-15, including epithelial cells, monocytes, macrophages, and ESCs, that can function to mediate NK cells activation, expansion, function and survival 141. Differentially, IL-15 plays a major role in recruiting unique CD16^-^NK cells with low cytotoxicity in the human endometrium. This is partly via selective extravasation of peripheral blood counterparts from the local microvascular circulation 142. Decreased autophagy levels in ESCs promotes cell viability and invasion, restricts cell apoptosis, and suppresses the activation of NK cells (low CD16, Granzyme B and IFN-γ) by upregulating IL-15Rα and IL-2Rβ, thus contributing to the growth and immunosurveillance of ectopic lesions 143. Importantly, impaired autophagy of ectopic ESCs mediated by estrogen dependence and progesterone resistance can lead to high levels of IL-8 and IL-23 secretion in a STAT3-HCK (hematopoietic cellular kinase)-dependent manner, which further promotes immunosurveillance of ectopic lesions by inducing the differentiation of COX-2^high^CD16^-^ NK cells with low cytotoxicity and high levels of IL-10 and TGF-β (**Figure 4**) 144. Additionally, IL-8 protects ectopic cells against death by apoptosis, and promotes cell adhesion and implantation in the pelvic cavity 145. Interestingly, treatment with rapamycin, an inhibitor of the mTOR pathway, decreases the levels of IL-15 receptors in ESCs by downregulating IL-15-mediated ESC growth and invasion 143, and restricts endometriosis development by inhibiting COX-2^high^CD16^-^ NK cell differentiation 144. These reports highlight the potential value of rapamycin in the treatment of endometriosis 143, 144, 146.

Notably, the elevated levels of peritoneal IL-8 due to defect autophagy enhance the FasL-induced apoptosis in activated T lymphocytes, contributing to the immune-privileged environment around the endometriotic implants and supporting their survival 145. Additionally, IL-8 exerts chemotactic activities primarily on neutrophils to further accelerate the development of endometriosis 147. A number of studies have shown that high levels of COX-2 are present in ectopic lesions and large amounts of PGE_2_ in PF 17. Considering the effects of COX-2 and PGE_2_ on promoting cell autophagy 69, 148, and the key roles of autophagy in M2 macrophage differentiation, it can be hypothesized that elevated M2 macrophages could be associated with COX-2/PGE_2_/autophagy signaling. These M2 macrophages can facilitate the remodeling of ECM and neovascularization of lesions 149, 150.

Programmed cell death 4 (PDCD4), a well-documented tumor suppressor, could inhibit tumor cell proliferation, migration and invasion at the transcriptional and translational levels 151. It has been shown that their expression was downregulated in the eutopic and ectopic endometrium from patients with endometriosis compared with normal endometrium. But its expression in the proliferative phase was comparable to that in the secretory phase. While in normal endometrium, their expression was significantly decreased in the progesterone-predominated secretory ESCs compared with that in estrogen-predominated proliferative phase in the proliferative phase was comparable to that in the secretory phase 152. PDCD4 inhibits the proliferation and colony-forming ability of endometrial cells via the NF-κB/MMP2/MMP9 signaling pathway, resulting in a reduction in autophagy-dependent degradation of proteins and programmed cell death, as well as promoting the survival of ectopic lesions 152. However, owing to the limited number of substantial clinical data, the role of autophagy in the pathogenesis of endometriosis remains controversial 132, 153. Based on the reports discussed above, we believe that the role of the estrogen/progesterone-autophagy-immunity axis in endometriosis should be better emphasized. Additionally, the molecular mechanisms involving these pathways require further study.

## Clinical implications and future perspectives

As discussed previously, the estrogen/ progesterone-autophagy-immunity axis is involved in the shedding of the endometrium, tissue regeneration and the defense mechanism during menstruation. Aberrant levels of autophagy are responsible for menstrual-related and endometrium-related diseases 154. Under the regulation of high levels of estrogen and progesterone resistance, reduced autophagy in ESCs can be combined with dysregulated immune responses to accelerate the development of endometriosis. Theoretically, we postulate the potential risk of endometriosis by detecting autophagy levels, subsets and phenotypes of immune cells in exfoliated endometrium in menstrual tissues, especially in patients with abnormal bleeding, extended menstrual periods, or abnormal endometrial thickness. Herein, we may possibly establish an immunological classification and evaluate its potential value in predicting the recurrence of endometriosis and other endometrial-related diseases (e.g., UCEC) by analyzing the autophagy levels and the phenotype subsets of immune cell in endometrial lesions and/or PF, as well as their genetic characteristics. Besides endometriosis, the dysfunctional autophagy can also contribute to a variety of other diseases, including cardiovascular disease 155, diabetes 156 and hepatocellular carcinoma 157). Several synthetic autophagy modulators have been identified as promising candidates for the treatment of cancers or benign lesions 158. Notably, rapamycin has been shown to protect spontaneous miscarriage by inducing decidual stromal cell autophagy-mediated decidual NK cell residence 87. Similarly, PPD also has therapeutic potential in endometriosis and UCEC by inducing cell autophagy 27, 38. Therefore, prospective studies will be needed to assess their potential values in early warning, diagnosis, recurrence risk prediction and personalized therapy for endometriosis and other endometrial diseases.

Given that autophagy is a dynamic process, static measurements could lead to misinterpretation of the result. Additionally, studies on the mechanism of menstruation are limited, due to the lack of suitable animal models (menstruation does not take place in rodents) and *in vivo* interventions in humans is not plausible. Thus, further well-designed studies would be required to fully understand the mechanisms underlying the pleiotropic roles of this axis in order to enrich our knowledge of physiological mechanisms associated with menstruation and pathological events such as menstrual dysfunction, endometriosis, and miscarriage.

## Abbreviations

AA: arachidonic acid
AMP: adenosine monophosphate
AMPK: adenosine 5'-monophosphate (AMP)-activated protein kinase
AP1: activator protein 1
Atg: autophagy-related
ATP: adenosine triphosphate
COX2: cyclooxygenase-2
CREB: cAMP responsive element binding protein
CRTC2: CREB-regulated transcription coactivator 2
CSF1: colony-stimulating factor 1
CXCL8: neutrophil chemotactic factor
ECM: extracellular matrix
EPC: endometrial glandular epithelial cells
ER: estrogen receptor
ESC: endometrial stromal cells
FIP200: focal adhesion kinase family interacting protein of 200kDA
FGF: fibroblast growth factors
FSH: follicle-stimulating hormone
GM-CSF: granulocyte-macrophage colony- stimulating factor
GPER: G-protein coupled estrogen receptor
HIF-1: hypoxia-inducible factor-1
HVEM: herpesvirus entry mediator
IGFR: insulin like growth factor receptor
IL-8: interleukin-8
JNK: c-Jun N-terminal kinase
LC3‑II: microtubule‑associated protein 1 light chain 3
LIF: leukemia inhibitory factor
MCP-1: monocyte chemoattractant protein-1
MITF: melanocyte inducing transcription factor
MMP: matrix metalloproteinases
mTOR: mammalian target of rapamycin complex
mTORC1: mTOR complex 1
NF-κB: nuclear factor kappa-B
PE: phosphatidylethanolamine
PDCD4: Programmed cell death 4
PDGF: platelet-derived growth factor
PF: peritoneal fluid
PGs: prostaglandins
PGE2: prostaglandin E2
PGF2α: prostaglandin F2α
PI3K: class I phosphatidylinositol 3-kinase
PPD: protopanaxadiol
PGR: progesterone receptors
PGRMC1: progesterone receptor membrane component 1
proLC3: LC3 precursor
ROS: reactive oxygen species
TFEB: transcription factor EB
TGF: transforming growth factor
TIMPS: tissue inhibitors of metalloproteinases
TSC: tuberous sclerosis complex
UCEC: uterine corpus endometrial carcinoma cells
ULK1: unc-51-like kinase 1
uPA: urokinase-type plasminogen activator
uNK: uterine NK cells
VEGF: vascular endothelial growth factor
